# Case of Myeleuthitis Hæmeuthyodes Encephalica, or Æsthesitis Encephalica

**Published:** 1819-11

**Authors:** T. Parkinson


FOR THE LONDON MEDICAL AND PHYSICAL JOURNAL.

### *Case of Myeleuthitis Hæmeuthyodes Encephalica, or Æsthesitis Encephalica.* By T. Parkinson, M.D.

*HISTORY.—Aug.* 21, 1819. I was called about noon to Mr. Wm. S. supposed to be deranged in his mind. He had returned the same morning from a journey to Bath and several other places, in the regular course of his business. At Bath he associated with some of his customers, and drank spirits with them in great excess for some days, contending vehemently with them on religious topics. On leaving Bath he became temperate in drinking,—indeed, abstemious. I have not been able to learn what business he transacted on his way to Redford, in Nottinghamshire, where he was seen by one of his acquaintances in an evident state of derangement of his mind. He had engaged his place on the outside of the coach to London, and had paid his fare from Leeds; but the guard of the coach strongly objected to taking him, on account of his mental derangement. His acquaintance, however, prevailed upon the

---

guard to let him proceed; and he reached the Saracen's Head Inn, London, in the forenoon of the 21st of August. A gentleman, travelling by the same coach, observed his incapability of conducting himself properly, and kindly looked well after his manners; and, having obtained his address, he very properly sent for Mrs. S. and she with difficulty escorted him to his home. I was immediately sent for, and attended him as soon as possible.

*Symptoms and Treatment.—Aug.* 21, *noon.* Exceedingly agitated; apprehending he was an apostate, and that he was called for to immediate and eternal punishment, seeing demons, as he imagined, surrounding him, to seize him and carry him to judgment. Remonstrances were not availing. His pulse was slow and contracted, his skin hot, his tongue white, his face flushed, his eyebrows knitted, his voice loud and harsh, his language imperative. His persuasion that all he imagined was real, could not be weakened by any means that could be employed. Ungovernable in his actions; bent upon self-destruction; begged for others to destroy him.

Fiat venæsectio statim, et detrahantur unciæ viginti sanguinis, saltem e bractio.

℞. Hydrarg. subm. gr. vj. cons. rosæ canin. q. s. ft. bolus, statim post venesectionem sumendus, super lactando cochl. larg. iv. solutionis insequentis hora 3tia quaque donec alvus copiose soluta sit.

℞. Magn. sulph. ℥ ij. aquæ commun. ℥ vij. M ft. solutio.

Applicatur, aqua frigida capiti assidue.

3 P.M.—Somewhat more tranquil. Face less flushed; pupils contracted, eyes fierce; voice and mind nearly the same. Pulse more dilated and quicker. Medicine operated well.

Rep. VS. ad ℥xx.

℞. Antimon. tart. gr. ij. sacch. alb. ʒij. potassæ supertart. ʒſs. aquæ com. ℥vij ſs. M. Sumat cochl. larg. iij horâ 3tia quaque.

℞. Ung. hydrarg. fort. ʒj. intern. femorum, &c. assidue applic. horâ 4ta quaque. Total abstinence from any thing but water.

*22d,* 9 A.M.—Symptoms of the same quality, though less violent.

6 P.M.—Copious alvine evacuations. Very feeble, and much stiller. Three stools.

Straight waistcoat. VS. ad. ℥xvj.

Empl. lyttæ toti capiti. Persistat in usu ung. hydrargyr. et mistur. antimon.

*23d.*—Free alvine discharge; but less quiet. Ungovernable; face flushed; pulse quick and hard.

Persistat in usu medic. Applicantur sanguisugæ xij. temporibus.

6 P.M.—Much better. Leeches bled well; copious alvine discharges, dark-coloured and watery.

Rep. empl. lyttæ capiti. Persistat in usu ungt. hydrargyri.

9 P.M.—Pulse feeble; general symptoms less violent.

℞. Assafœtid., ammon. carbon., camph. an. gr. iij. f. bolus.

℞. Infus. gentianæ, ℥vij ſs. sp. æther sulph. ʒ ij. M. capiat bolum, et cochl. larg. iij. Misturæ horis 4tis.

24*th*, 9 A.M.—Slept at intervals; in all, about two hours. No evacuations. Pulse too full, and quick. Evident remissions. The exacerbations at nine in the morning and at nine at night.

Applic. sanguisugæ xij. temporibus.

℞. Hydrarg. subm. gr. xij. extract. colocynth, ʒj. in pilul. xvj. div. sumat. iv. horâ 3tia quaque donec alvus soluta sit.

℞. Solut. magn. sulph. ℔ ſs. Sumat cochl. larg. iij. post singul. dosin pilularum.

6 P.M.—Leeches bled freely; medicine operated very copiously. Much calmer and cooler.

Persistat in usu solut. magn. sulphat.

25*th*, 9 A.M.—Very copious discharge by stool; pulse and countenance much better; intervals of refreshing sleep; evident intermission. The paroxysm coming on about 9 P.M.

A saline mixture, with antimonial wine.

Meet the next paroxysm, which is predicted at nine to-morrow morning.

26*th*, 9 A.M.—Has had some sleep at intervals; has been more tranquil. About nine, paroxysm and return of all the symptoms in excess. Pulse 130; ungovernable.

Detrahantur sanguinis unciæ xx. saltem e nucha colli statim cucurbitalis, et repetatur operatio vespere si paroxymus recurrit.

Applicantur sinapismi pedibus ambobus.

No evening paroxysm. Copious purging; mouth sore with unguent hydrarg.

Solut. magnes. sulph. addendo syrup. rhamn. ℥j. Omitta ungt. hydrarg.

10 P.M.—Slept longer at a time.

Persistat in usu solut. magn. sulph.

27*th*, 10 A.M.—Copious evacuations; slept nearly two hours. Began about nine to be worse. Pulse quickened, 130; face flushed, eyes reddened; mind much deranged. Sinapisms had acted well.

As the paroxysm would begin to decline about noon, capiat pulverem sequentem horâ 1ma. P.M.

℞. Pulv. ipecacuanhæ comp. ℈j. ft. pulvis.*

---

* Contains two grains of opium. This note is for our foreign readers.—EDIT.

9 P.M.—Had become more calm about one; took the powder, and slept more than three hours.

Repitatur pulv. ipecac. comp. ℈j. horâ prima mane.

28*th*, 10 A.M.—Has had three stools: all the stools have been dark-coloured and watery hitherto, but now bilious. The first dose of opiate calmed, the last laid prostrate. Sweats profusely.

4 P.M.—Stiller, more rational; pulse feeble; mouth very sore.

No medicine. Wine and water, or a little porter; beef-tea, gruel, tea, or milk-porridge.

29*th*, 10 A.M.—Much more tranquil and rational. Pulse soft and much slower; slept soundly more than two hours at a time; no stool.

Capiat dosin solutionis magn. sulph. et bibat ad libitum infusi rosæ. Change of linen, washing, &c.

3 P.M.—Bore shifting well. In all respects better; takes wine and water, milk, broth, &c.

Persistat in usu solutionis magn. sulph. et infusi rosæ.

30*th*.—Better; voice changed from harsh to soft-toned; from imperative to precative. Persistat in usu medicamentorum.

31*st to Sept.* 3*d*.—Rapid amendment.

4*th*.—Convalescent. Ordered into the country.

I consider the case here related to have been inflammation of the substance of the brain; and that the means employed, and to the full extent mentioned, were not only efficacious in preserving the life and the intellect of the patient, but were probably such only as would have been availing.

I have publicly given it as my opinion, and have taught it to my pupils, that the brain within the head is an organ of sense, composed of the same materials and association as all the perceiving organs, such as the retina, &c.; viz. *myeleuthitona hæmeuthytodes*,—an association of muscle with brain: otherwise I cannot conceive how it could obtain the function called perception, consisting of the two essential attributes of animal life, sensation and motion. (Vide my *Synopsis Zoo-Nosologiæ.*) But the great office of the brain within the head is to elaborate perceptions, obtained by the organs of sense, into judgment or mind; and to direct the commands of the mind, *volitions*, through the nerves, *myeleuthytona enyphantodes*, to the moving solids, *hæmeuthytona*, vulgarly called muscle, from some supposed resemblance to a mouse.

The function of the brain, then, being *æsthesis*, when it is in hyperfunction, as it must be under inflammation, the elaboration of perceptions into mind must be incorrect, and the volitions indiscreet: these are the very symptoms which characterize inflammation of the brain, and are the inevitable consequences of it. As for fever, it is symptomatic, and symptomatic only, of local inflammation; it therefore does not admit of any distinct appellation. Inflammation of the first order of solids, in its progress, always must be accompanied with a corresponding degree of hyperbiosis throughout the whole of that genus of solid which is the seat of the original attack; for example, the case before us, *myeleuthitis encephalica hæmeuthytodes*,—that is, an inflamed state of the brain within the head,—had its concomitants; namely, hyperfunction of all the organs of sense: the patient's eyes, ears, and all the other perceiving organs, even the rete mucosum, the general perceiving organ, all were in excessive exercise of their natural function, marked and known by the perceptions being incorrect and excessive. Had the seat of the original attack been in a membranous solid, as it is called, it would have been known by a very different assemblage of symptoms; for, the function of membrane being *parenthesis*, the congress of symptoms would constitute *parenthitis*, or inordinate and painful interposition between one solid and another. The extension of inflammation would have been especially in membranes, and have been shown by the general constitutional sympathies. Fever, if the term must be mentioned again, is not the *idiopathic* affection, but is *symptomatic*, and is a necessary and inevitable effect of a certain degree of intensity and extension of definable local inflammation. Examine inflammation of the muscle, *rheumatismus, hæmeuthitis*; the identity of the disease is known by different symptoms to either of the before-mentioned affections, though the precise nature of the affection is the same in them all; namely, exercise of their natural functions above the limits of health, in the *rheumatismus kinesitis*, or inordinate and painful muscular action. I shall conclude by observing, that inflammation of any genus will always have certain peculiar marks, by which, with an ordinary share of discernment and attention, it may be distinguished from inflammation of any other genus of solid.

